# Registries in allergy: Structure, target groups, and key findings of allergy-focused registries in Germany 

**DOI:** 10.5414/ALX02536E

**Published:** 2024-12-31

**Authors:** Marie Farkic, Philipp Globig, Aikaterina Alexiou, Margitta Worm

**Affiliations:** Division of Allergy and Immunology, Department of Dermatology, Venerology and Allergology, Charité – Universitätsmedizin Berlin, Berlin, Germany

**Keywords:** registry, allergology, anaphylaxis, severe asthma, contact allergy, atopic dermatitis, biologics, real-world data

## Abstract

In allergology, clinical registries fill knowledge gaps of epidemiology, mechanisms of allergic diseases, and real-world treatment outcomes. Considering the continuous rise of allergic diseases worldwide, registries become increasingly important for the optimization and harmonization of patient care. In the current review, we present four ongoing allergy-focused registries initiated in Germany. We conducted a focused literature search and discussed their structure, main purposes, and findings. Registries included are the “Information Network of Departments of Dermatology”, the “European Anaphylaxis Registry”, the “GAN Severe Asthma Registry”, and “TREATgermany”. Despite differences in scope and operation, all registries gather harmonized real-world data that is indispensable for evidence-based decision making in clinical practice and ultimately improves patient care in allergology.

## Introduction 

Allergic diseases have become a global health issue and heavily impact healthcare resources as they continue to increase worldwide [1, 2]. In Germany, almost one third of the adult population was diagnosed with at least one allergic disease in their lifetime [3]. Considering the high prevalence of allergies in the German population, but also alarming upward trend worldwide [1, 2, 4], registries play an increasingly important role in the surveillance and management of allergic diseases. 

They provide comprehensive clinical data to different stakeholders (healthcare professionals, regulatory bodies, patients) [4, 5] and may cover the following important aspects: 

First, registries enable the evaluation of real-world (long-term) effectiveness and safety of drugs that are implemented in routine care. Randomized controlled trials (RCT) consist of a homogenous, narrowly defined study cohort in accordance with predefined inclusion criteria. Registries include a broader spectrum of participants, leading to an increased generalizability of gathered data [5]. Such registries in allergology are “The GAN Severe Asthma Registry” for severe asthma and “TREATgermany” for atopic dermatitis (AD). 

Second, registries generate important evidence for treatment guidelines where clinical trials are difficult to conduct. Additionally, they facilitate the surveillance of disease management in accordance with current guideline recommendations [5]. Considering the unpredictability and severity of anaphylaxis [6, 7, 8, 9] and lack of robust evidence for treatment guidelines [10, 11], findings of the “European Anaphylaxis Registry” (EAR) are essential. 

Lastly, registries may also serve as a tool for active surveillance of allergies [5]. By continuously analyzing epidemiologic data, novel trends can be detected and used to implement preventive measures by regulatory bodies. The “Information Network of Departments of Dermatology” (“Informationsverbund Dermatologischer Kliniken” – IVDK) actively monitors contact allergies in the population of German-speaking countries [12]. 

**Figure 1:**
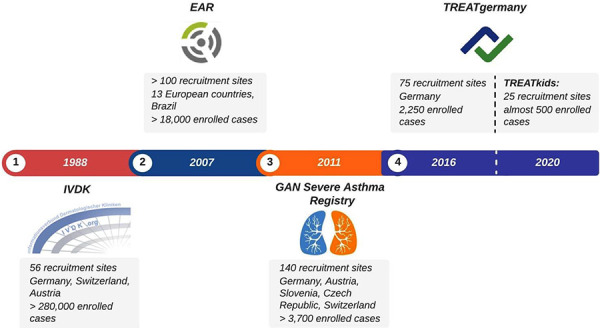
Presented registries and year of their establishment.

Here, we present the ongoing allergy-related registries initiated in Germany (Figure 1) and discuss their main purposes, findings, strengths, and limitations. 

## Materials and methods 

We analyzed the structure and key findings of the different registries covering topics of allergology. A focused literature search was performed, and the obtained data were critically reviewed. Search was conducted using PubMed database as well as the registries’ websites. 

## Results 

Structure, target groups, and recent data from the allergy registries 

### The Information Network of Departments of Dermatology (IVDK) 

The IVDK is a network of 56 currently active dermatology departments across Germany, Austria, and Switzerland, initiated in 1988, that continuously monitors the prevalence and trends of contact allergies in the population [12]. Generation of data does not depend on the selective recruitment of patients, as all routinely patch-tested patients in cooperating centers for suspected contact allergy are included [12]. Patch testing is performed in a standardized manner according to national and international guidelines using test preparations of the “Deutsche Kontaktallergie-Gruppe”, to which all participating centers belong [42] (Table 1). 

The IVDK has published various analyses of long-term surveillance periods, regarding the sensitization patterns in the overall population, but also considering specific allergens and distinct demographic subgroups. Assessment of current trends and sensitization pattern of contact allergies are presented in Table 2.

### Evaluation of regulatory actions 

Continuous, long-term surveillance of sensitization patterns enables the detection of trends, emerging allergens, persisting problems and the evaluation of the success of implemented directives [12]. Epidemiological data gathered by the IVDK illustrated the success of regulatory measures counteracting the “epidemic” of contact allergies to the preservative methylisothiazolinone (MI) in Europe [21, 22]. As an example of detecting persistent problems, IVDK data revealed a consistently high prevalence of nickel allergy in young female patients, despite the first EU nickel directive [23]. Investigations initiated by the IVDK exposed non-compliance with the first nickel directive, which was revised in 2004 [24]. Consequently, sensitization to nickel in young females significantly decreased from 2005 to 2012 [24]. 

### Occupational dermatitis 

The IVDK also plays an essential role for the identification of occupations at risk for the development of occupational dermatitis due to an increased allergen exposure [12]. 

### The European Anaphylaxis Registry (EAR) 

The EAR was launched in 2007, started recruiting patients of German speaking countries and expanded to other European countries in 2011 [13]. To date, the registry encompasses over 150 specialized tertiary allergy centers, 43 of which are located in Germany [43]. Participating centers continuously collect real-life data from patients of all age groups who suffered from anaphylaxis ≤ 12 months prior to their visit [13] (Table 1). 

The spectrum of eliciting allergens and clinical presentation differ significantly between children and adults [25, 26, 27]. Risk factors of severe anaphylaxis as observed by the EAR are presented in Table 2. 

### Discrepancy of guideline recommendations and real-life data regarding the use of epinephrine as the first line drug in anaphylaxis 

Registry data repeatedly reveal an underuse of epinephrine as the first-line treatment in anaphylaxis (23.2% of cases between 2006 and 2017) [11, 25, 27]. Epinephrine administration by health professionals varied internationally, being lowest in Germany (19.6%), while overall increasing within the analyzed period, indicating an improved guidelines awareness and adherence [11]. Additionally, data on adrenaline autoinjector prescription practices reveal a low adherence to the current “European Academy of Allergy and Clinical Immunology” guidelines outside of specialized allergy centers, underpinning the relevance of allergy centers for ensuring adequate patient care [28]. 

### The GAN Severe Asthma Registry 

The GAN Severe Asthma Registry intends to provide data on the epidemiology, phenotypes, and treatment of severe asthma [29]. It aims to close important knowledge gaps regarding pathomechanisms, natural course, and prognosis of the disease [41]. The registry was established in 2011 by the “German Asthma Net e.V.” (GAN) [41] and started the recruitment of patients ≥ 6 years suffering from physician-diagnosed severe asthma. Currently, the registry encompasses 140 participating centers mainly from Germany [41] (Table 1). 

### Characterization of the severe asthma cohort 

Analysis of baseline characteristics of patients enrolled in the GAN Severe Asthma Registry demonstrates the heterogeneity of severe asthma. Despite being treated in specialized clinics or practices and by specialized pulmonologists, data of the registry reveal a suboptimal disease control in severe asthma patients [16, 19]. Continuous measurement of functional parameters and biomarkers of patients aids the characterization and differentiation of asthma phenotypes, facilitating the identification of patient subgroups that will respond to a given treatment regimen [29] (Table 2). 

### Real-world practice, effectiveness, and safety of biologics in patients with severe asthma 

Comparative assessment of clinical response in patients receiving biologics and those without targeted therapy, reveal higher response rates and more frequent disease remission in patients treated with biologics [30] (Table 2). Treatment with mepolizumab results in the improvement and long-term stability of asthma control and lung function [31]. 

### The TREATgermany registry 

TREATgermany is a multicenter, observational registry and currently encompasses ~ 75 participating German centers [44]. Data of patients with moderate-to-severe AD is prospectively collected in a real-world setting [20]. Considering the rapid progress of targeted systemic treatment for AD in the past years, TREATgermany independently and continuously evaluates systemic treatment in routine care [32, 33]. The registry not only examines drug safety and effectiveness of biologics (dupilumab, tralokinumab, lebrikizumab) but also of JAK inhibitors, such as baricitinib [34]. In 2020, the section TREATkids was launched [14] and currently comprises almost 500 children and adolescents under the age of 18 years [44]. 

### Real-world effectiveness and safety of biologics 

Treatment with dupilumab significantly improves objective parameters and PROs compared to baseline values. Dupilumab displays a favorable safety profile, and much lower discontinuation rates compared to cyclosporin A (CyA) [35]. However, analysis of TREATgermany data reveals more dupilumab-related ocular complaints (29.8% of patients) than preceding trials [35] (Table 2). Dupilumab induces a shift of the skin microbiome of patients toward the skin flora of healthy controls, largely independent of clinical response [36]. 

## Discussion 

Participating centers of all registries gather data in a standardized manner and transmit them to the registry centers for data analysis. In general, standardized data acquisition results in a large set of harmonized data with increased statistical power. This enables stratification, subgroup analysis, and even interregional and -national comparisons. Furthermore, continuous exchange with participating centers is fundamental for the harmonization of disease management in accordance with current guidelines. Because the registries focus on different allergic diseases, they vary in scope and operation. However, all the registries assess real-world data and therefore provide valuable insight into the current state of patient and disease management and draw attention to gaps in patient care. The IVDK serves as allergen surveillance systems and not only addresses clinicians but also regulatory bodies for the implementation of preventive measures and directives [12]. Data of the EAR repeatedly highlighted the need for an increased guideline awareness and patient education regarding the management of anaphylaxis [11]. Real-world data obtained by the GAN Severe Asthma Registry and TREATgermany regarding the (long-term) effectiveness and drug safety of novel targeted therapies is indispensable. The observed disparities of dupilumab-induced ocular complaints in real-world patients compared to participants in clinical trials underpin the relevance of registries in identifying and understanding product safety in the diverse cohort of routine care [5, 37]. The heterogeneity of patients with severe asthma limits the generalizability of drug effectiveness observed in well-defined study cohorts of clinical trials. The eligibility criteria for many RCTs would exclude 95% of asthma patients [38]. Registries like the GAN Severe Asthma Registry and TREATgermany will continue to provide crucial information to fill gaps for decision-makers by assessing real-world drug safety and effectiveness. Furthermore, assessment of biomarkers and research on underlying disease mechanisms and endotypes, will pave the way for an individualized treatment approach [39]. Findings of the presented registries are limited by only voluntary participation of recruitment centers and patients, which may introduce selection bias [25, 27, 31, 40]. Hence, apart from data of the IVDK, they may not be representable for the population as total and epidemiological studies are not possible. 

## Conclusion 

In allergology, registries act as allergen surveillance systems by actively monitoring trends and emerging elicitors of contact allergies and anaphylaxis in the population. Moreover, registries give insight into real-world long-term safety and effectiveness of systemic therapies for AD and asthma and significantly contribute to patient-oriented research. Therefore, they should be established and interconnected for all allergic diseases. 

## Authors’ contributions 

MF performed the literature search, drafted and wrote the manuscript. PG and AA revised the manuscript. MW was responsible for the concept and revising of the manuscript. All authors reviewed and approved the final version of the manuscript and its submission. 

## Funding 

None. 

## Conflict of interest 

Prof. Worm declares the receipt of honoraria or consultation fees by the following companies: Novartis Pharma GmbH, Sanofi-Aventis Deutschland GmbH, DBV Technologies S.A, Aimmune Therapeutics UK Limited, Leo Pharma GmbH, AstraZenceca GmbH, ALK-Abelló Arzneimittel GmbH, Lilly Deutschland GmbH, Kymab Limited, Amgen GmbH, Abbvie Deutschland GmbH & Co. KG, Pfizer Pharma GmbH, Mylan Germany GmbH (A Viatris Company), Boehringer Ingelheim Pharma GmbH & Co. KG, GlaxoSmithKline GmbH & Co. KG, Almirall S. A., Amgen GmbH, Pfizer Deutschland GmbH, Bristol-Myers Squibb GmbH & Co. KGaA and FomF GmbH. 

All other authors declare to have no conflict of interest. 

**Table 1.** Overview of the registries.

	**IVDK**	**EAR**	**GAN Severe Asthma Registry **	**TREATgermany ** **TREATkids**
Year established	1988 [12]	2007 [13]	2011 [41]	2016 [41] 2020 [14]
Countries	Germany, Switzerland, Austria [15]	13 European countries and Brazil [43]	Germany, Austria, Slovenia, Czech Republic, Switzerland [41]	Germany
Participating centers	Dermatology dpts [12]	Tertiary allergy centers	Pulmonology departments and practices with specialist pulmonologists [16]	Dermatology dpts and practices [17, 18]
Number of participating centers	56 [15]	> 100 [13] Germany: 43 centers	140, 73 % German centers [41]	Approximately 75 [44] Approximately 25 [44]
Inclusion criteria	Patch tested patients for suspected contact allergy [12]	Anaphylactic reaction ≤ 12 months prior to enrollment [13]	Physician-diagnosed severe asthma (according to the ERS/ATS^2^ criteria) [19]	Moderate-to-severe atopic dermatitis: oSCORAD > 20 or systemic treatment current or ≤ 24 months prior to enrollment [44]
Included age	All	All	≥ 6 years [16]	≥ 18 years [44] < 18 years [44]
Enrolled cases	> 280,000 [42]	Total: > 18,000 Germany: 10,140 [43]	> 3,700 [41]	2,250 [44] almost 500 [44]
Follow-up visits	–	–	At least once a year ideally for 15 years [16]	Every 3 – 6 months for ≥ 24 months [20]
Gathered data	Patient demographics, medical history			
	Results of patch tests, suspected allergen source and occupational context, anatomical site of the dermatosis, cofactors [12, 42]	Detailed description of anaphylactic reaction and management [13]	Current asthma treatment Lung function, biomarkers, exacerbations, asthma control (GINA^2^ control status) Patient-reported outcomes (PROs) [16]	Current AD treatment Objective parameters: IGA; oSCORAD, EASI^3 ^ Patient-reported outcomes (PROs) Biosamples (optional) [44]

^1^European Respiratory Society/American Thoracic Society; ^2^Global Initiative for Asthma; ^3^Investigator global assessment, objective scoring atopic dermatitis, eczema area and severity index.

**Table 2.** Selected key findings of the presented registries.

**Registry & [Reference]**	**Key findings**
IVDK [22] Study period: 2007 – 2018 Patients: n = 125,436	Time trends and current spectrum of CA to the most common allergens (DKG baseline series) (excerpt)
	Most common allergens and overall percentage of positive test results: nickel (14.7%), fragrance mix I (8.1%), myroxylon pereirae resin (7.5%), cobalt (5.2%), no definite trend **Preservatives: ** - MI epidemic: 2014 peak in Europe followed by a continuous decline - 2018: almost pre-epidemic level of MCI/MI CA reached **Propolis:** - Significant increase from 2.35% positive tests in 2007 – 2010 to 3.94% positive tests in 2015 – 2018
EAR [9] Study period: 2007 – 2017 Patients: n = 7,316	Risk factors of severe anaphylaxis:
	- Higher age - Mastocytosis - Vigorous physical exercise (independent from elicitor) - Male sex - Psychological burden - Intake of beta-blockers or ACE inhibitors
GAN Severe Asthma Registry [30] Registry data up to July 2022 Patients: n = 443	Treatment response to biologics according to BARS(-L) criteria (Milger et al. [19])^1^. Comparison of treatment with and without biologics.
	One year of treatment with biologics: - **Greater improvements regarding** annual exacerbations, oral corticosteroid dose reduction, ACT score, FEV_1 _ - Higher BARS response rates: “good” in 61.4 vs. 34.8% (biologic treatment vs. no biologics) - **Higher remission rates:** 37.6 vs. 17.2% (biologic treatment vs. no biologics)
TREATgermany [32] Registry data up to July 2021 Patients: Dupilumab n = 369 CyA n = 41	Real-world effectiveness and drug safety of dupilumab and CyA
	Dupilumab: favorable safety profile, robust long-term effectiveness CyA: higher discontinuation rates - After 12 months: 78% (CyA) vs. 5% (dupilumab) - Reasons for discontinuing CyA: side-effects (31%), insufficient efficacy (27%), no report (56%) Most frequent dupilumab-associated adverse events: - Ocular complaints (29.8%), predominantly conjunctivitis

^1^Biologics Asthma Response Score [30]. CA = contact allergy; DKG = German Contact Dermatitis Research Group (Deutsche Kontaktallergie-Gruppe); MI = methylisothiazolinone; MCI = methylchloroisothiazolinone; ACE = angiotensin converting enzyme; BARS = Biologics Asthma Response Score; ACT = asthma control test; FEV_1_ = forced expiratory volume in 1 second; CyA = cyclosporin A.
